# Identification of a New Antibacterial Sulfur Compound from* Raphanus sativus* Seeds

**DOI:** 10.1155/2016/9271285

**Published:** 2016-10-03

**Authors:** Jeries Jadoun, Ahmad Yazbak, Salwa Rushrush, Amira Rudy, Hassan Azaizeh

**Affiliations:** ^1^The Galilee Society Institute of Applied Research, Affiliated to University of Haifa, P.O. Box 437, 20200 Shefa-‘Amr, Israel; ^2^Ben-Gurion University of the Negev, P.O. Box 653, 8410501 Beer Sheva, Israel; ^3^Tel-Hai College, 12208 Upper Galilee, Israel

## Abstract

*Raphanus sativus* L. (radish), a member of Brassicaceae, is widely used in traditional medicine in various cultures for treatment of several diseases and disorders associated with microbial infections. The antibacterial activity of the different plant parts has been mainly attributed to several isothiocyanate (ITC) compounds. However, the low correlation between the ITC content and antibacterial activity suggests the involvement of other unknown compounds. The objective of this study was to investigate the antibacterial potential of red radish seeds and identify the active compounds. A crude ethanol seed extract was prepared and its antibacterial activity was tested against five medically important bacteria. The ethanol extract significantly inhibited the growth of all tested strains. However, the inhibitory effect was more pronounced against* Streptococcus pyogenes* and* Escherichia coli*. Bioassay-guided fractionation of the ethanol extract followed by HPLC, ^1^H-NMR, ^13^C-NMR, ^15^N-NMR, and HMBC analysis revealed that the active fraction consisted of a single new compound identified as [5-methylsulfinyl-1-(4-methylsulfinyl-but-3-enyl)-pent-4-enylidene]-sulfamic acid, which consisted of two identical sulfur side chains similar to those found in ITCs. The minimal inhibitory concentration values of the isolated compound were in the range of 0.5–1 mg/mL. These results further highlight the role of radish as a rich source of antibacterial compounds.

## 1. Introduction

*Raphanus sativus* L. (radish), a member of the cruciferous family, is an annual herb consumed as vegetable throughout the world. Various varieties are available that differ mainly in the size, shape, and color of their thick roots [1]. The roots are the most valuable and edible part of radish, although the stem and leaves have been also used for food flavoring or preservation [2]. As similar to other cruciferous vegetables, the nutritional value of radish is derived from its content of many essential minerals and vitamins, carbohydrates, high content of fiber, and low content of fat [3]. Radish has also valuable medicinal properties. It is widely used in traditional medicine in various parts of the world for treatment of different ailments and disorders affecting the respiratory, urinary, and gastrointestinal systems, anemia, female and male infertility, and the skin [1, 4]. Its leaves and roots are also used as antimicrobial agents [1, 5]. Many of the pharmacological activities of radish are attributed to the occurrence of a wide range of secondary metabolites, including alkaloids, phenolics, flavonoids (including anthocyanins), coumarins, carotenoids, antioxidant enzymes, terpenes, glucosinolates, and other compounds [1, 3, 5–9].

The application of radish in traditional medicine to treat various infectious diseases has stimulated a great interest in investigating its antimicrobial activity. Various studies have demonstrated the ability of different plant part extracts to suppress growth of a wide range of bacterial strains including drug-resistant ones. Radish root juice can inhibit the growth of* Escherichia coli*,* Pseudomonas aeruginosa*,* Pseudomonas pyocyaneus*,* Salmonella typhi*,* Klebsiella pneumoniae*,* Bacillus subtilis*,* Staphylococcus aureus*, and* Enterococcus faecalis* [10]. Crude aqueous and other solvent extracts (including ethanol, methanol, ethylacetate, and petroleum ether) of black radish root peels display inhibitory effect against* S. aureus*,* Micrococcus luteus*,* E. coli*,* S. typhi*,* P. aeruginosa*,* Bordetella bronchiseptica*,* B. subtilis*,* K. pneumonia,* and* Enterobacter aerogenes* [11]. Methanol extracts of white and black peel taproots can inhibit a wide variety of food-associated pathogens, such as* Arthrobacter atrocyaneus*,* Corynebacterium ammoniagenes*,* Enterobacter hormaechei*,* Kocuria rosea*,* Neisseria subava*,* Pantoea agglomerans*,* Proteus vulgaris*,* Psychrobacter immobilis*,* Shigella dysenteriae*,* B. sphaericus,* and* Corynebacterium flavescens* [12]. Likewise, methanol, ethyl acetate, and chloroform extracts of the root, stem, and leaf of white radish exhibit significant antibacterial activity against various foodborne and drug-resistant pathogenic bacteria including* B. subtilis*,* E. coli*,* P. aeruginosa*,* S. aureus*,* S. epidermidis*,* E. faecalis*,* S. typhimurium*,* K. pneumoniae*,* E. aerogenes*, and clinical isolates of* E. cloacae* [2, 13]. Ethanol and methanol extracts of white radish seeds have been shown to be active against various pathogenic bacteria including* E. coli*,* K. pneumoniae*,* P. vulgaris*,* P. aeruginosa*,* S. aureus*,* S. sonnei*,* S. typhi,* and* S. paratyphi *[14]. Methanol and aqueous seed extracts have been also reported to have antimicrobial effect against various plant pathogenic bacteria and fungi [15].

As a member of the Cruciferae family, radish is rich in glucosinolates, well-known biologically active and chemically diverse sulfur-containing compounds, which are the precursors of isothiocyanates (ITCs) [6]. Glucosinolates and ITCs are variably distributed in the different radish plant parts and among different radish cultivars [1, 16–18]. ITCs exhibit a wide antimicrobial activity affecting various food spoilage and pathogenic bacteria, including multi-drug-resistant strains [19, 20]. A correlation between ITCs and antibacterial activity has been found in radish root, stem, and leaf extracts. However, the correlation between the total ITC content and antibacterial activity was low [16]. A recent study demonstrated that sulforaphene, one of the major ITCs in radish seeds, possesses a broad and strong antibacterial activity against drug-resistant strains of* Helicobacter pylori* and* S. aureus* [21]. However, in this study no comparison was made between the activity of sulforaphene and the crude extract. In addition to ITCs, radish seeds contain various secondary metabolites including alkaloids, flavonoids, glycosides, phenols, sterols, and tannins [12, 14]. Many of these compounds are known to exert antimicrobial effects [22] and have also been implicated in the antibacterial activity of radish seed extracts [14]. Altogether, these findings imply that ITCs are not the only compounds responsible for the antibacterial activity of radish seeds.

In this study, an ethanol extract of cultivated red radish seeds was found to exhibit significant inhibitory activity against different bacterial strains. Therefore, the main objectives of the current study were to evaluate the antibacterial potential of the radish seed extract against different gram-positive and gram-negative bacteria and to establish a bioassay-guided fractionation approach for isolation and consequently identification of the bioactive ingredients in the seed extract. Using bioassay-guided fractionation followed by HPLC, ^1^H-NMR and ^13^C-NMR, ^15^N-NMR, and Heteronuclear Multiple-Bond Correlation (HMBC), a new antibacterial sulfur compound was identified.

## 2. Materials and Methods

### 2.1. Plant Material

Cultivated red radish organic seeds were obtained from the biotechnological greenhouse center of the Galilee society, where also a voucher seed specimen RS-110 has been deposited.

### 2.2. Bacterial Strains

The antibacterial activity was carried out by the gram-positive strains,* Streptococcus pyogenes* (ATCC 19615) and* S. aureus *(ATCC 25923), and gram-negative strains,* E. coli* (ATCC 25922),* S. typhimurium* (ATCC 14028), and* K. pneumoniae* (ATCC 700603). These reference strains were used as representatives for pathogenic bacteria. All strains were maintained on Tryptic Soy Broth (TSB) containing 20% glycerol and stored at −80°C. Subcultures were freshly prepared before use by inoculation of a loop of stored culture into 5 mL TSB tubes followed by overnight incubation at 37°C.

### 2.3. Preparation of Crude Extract from Radish Seeds

Twenty-five grams of seeds was ground into fine powder, suspended in 150 mL of ethanol (85%), and agitated at 150 rpm for 24 h at room temperature. The suspension was then filtered (using glass fibers and Whatman Number 3 filters) under vacuum to remove undissolved solids and evaporated to ca 1/15th of its original volume using rotary evaporator, at 40°C to remove the ethanol and to concentrate the dissolved compounds. The concentration of the total soluble compounds in the crude extract was determined by measuring the dry weight of a small aliquot of the extract dried at 65°C overnight.

### 2.4. Determination of the Antibacterial Activity

For determining the antibacterial activity of the extract/various fractions against the bacterial reference strains, the agar-well diffusion assay was used. Bacterial strains were grown overnight in 5 mL TSB, diluted by 1 : 100–1 : 500 to get a density of 0.5 McFarland standard (10^7^ ~ 10^8^ cfu/mL) with same medium, and then were evenly spread onto the surface of Tryptic Soy Agar (TSA) or THB agar plates using sterile swabs. Four to five wells (5 mm in diameter) were made in each plate with sterile Pasteur pipettes. About 80–100 *µ*L of crude extract containing 13–20 mg of soluble compounds was added to each well. For antibacterial activity screening of the fractions, 10 mg of each fraction was dissolved in 100 *µ*L of 15% methanol and loaded into the wells. Methanol (15%) and chloramphenicol (15 *µ*L of 10 mg/mL) were used as a negative control and positive controls, respectively. After allowing diffusion of the extract for 1 h at room temperature, the plates were incubated at 37°C for 24 h and then were observed for the presence of inhibition of bacterial growth as indicated by a clear zone around the wells. The sizes of the zones of inhibition were measured and the antibacterial activity was expressed in terms of the average diameter of the zone inhibition in millimeters. The absence of a zone inhibition was regarded as the absence of activity.

### 2.5. Determination of MIC

Fractions that demonstrated antibacterial activity were further analyzed by determining their minimal inhibitory concentrations (MIC) values. The MIC values of the fractions against the bacterial reference strains were determined by microdilution according to procedures developed by the National Committee of Clinical Laboratory Standards [23]. Prior to serial dilution with TSB or THB, each dried fraction was dissolved in 15% methanol. After transferring of 180 *μ*L from each dilution into wells of a 96-well plate (in triplicate), 20 *μ*L of fresh bacterial culture (grown as described above) was added to each well to obtain ca 5 × 10^5^ cfu/well and the microplates were then incubated at 37°C overnight. The MIC concentration was determined as the highest dilution of extract showing no detectable growth.

### 2.6. Fractionation, Purification, and Identification of the Active Compounds of the Ethanol Crude Seed Extract

Following ethanol evaporation, the crude extract was extracted using different solvents of increasing polarity, starting with hexane followed by ethyl acetate. The hexane, ethyl acetate, and the remaining aqueous fractions were tested for antimicrobial activity following solvent (or water) evaporation and dissolution in methanol as described above. The active fraction (ethyl acetate fraction) was dissolved in dichloromethane : methanol and further fractionated through Silica gel (Merck, 40–60 *μ*m) column chromatography. The separated compounds were sequentially eluted, while increasing the eluent polarity, starting with n-hexane, n-hexane : ethyl acetate at ratios 2 : 1, 1 : 1, and 1 : 2, dichloromethane, and 5, 15, and 20% methanol in dichloromethane. In order to determine the purity of the fraction, each of the obtained fractions was analyzed using silica gel thin layer chromatography (TLC) and reverse phase HPLC. Fractions showing similar compound composition were pooled. TLC analysis was performed using silica gel glass plates 60 F_254_ (Merck, 0.25 mm). HPLC analysis was carried out using HPLC (Agilent) equipped with UV detector and column chromatography [LiChroCART® 250-4 (Merck), RP-18 (Merck), 40% Acetonitrile; 235 nm]. The molecular mass of the isolated compound in the active fraction was determined using HPLC/MS and High Resolution Mass Spectroscopy (HRMS). Mass spectrometry was carried out with Waters LCT-Premier MS using Acetonitrile : H_2_O (75 : 25) as elution solvent and 0.25 mL/min solvent flow. The chemical structure of the active fraction was determined using ^1^H-NMR and ^13^C-NMR, ^1^H-^15^N HMBC, and ^1^H-^13^C HMBC. ^1^H-NMR and ^13^C-NMR were recorded in deuterated solvents on a Bruker 400 MHz spectrometer; chemical shifts were reported in parts per million.

## 3. Results and Discussion

### 3.1. Antibacterial Activity of the Radish Crude Seed Extract

Although the antimicrobial activity of radish has been investigated by several studies, only a few studies addressed the activity of radish seeds, particularly of cultivated red radish. In contrast to a previous study reporting the lack of activity of seed extracts (dichloromethane : methanol) against 14 different bacterial and fungal strains [24], our study demonstrated that ethanol crude seed extract was substantially active against all tested pathogenic bacterial strains (Table 1). The different findings between the two studies could be attributed to the different extraction methods (room temp. versus under reflux) or solvents applied for preparation of the extract, as demonstrated by several studies [11, 14, 25]. Jamuna et al. [25] found that, among the different extraction methods applied, cold extraction of fresh radish root was significantly better than dried cold extraction or soxhlet and in terms of antioxidant activities tested. Ahmad et al. [14] showed that the maximal antibacterial activity against eight different gram-negative and gram-positive bacterial strains was obtained with ethanol and methanol white radish seed extracts, compared to ethyl acetate, chloroform, benzene, and water. Janjua et al. [11] found that ethanol and ethyl acetate extracts of black radish-peels had the highest antimicrobial activity compared to other solvents, although the effect of these extracts was variable and species-dependent. The inhibitory zone range (11–20 mm) of the seed ethanol extract recorded in this study was comparable to that reported by Ahmad et al. [14] for ethanol and methanol seed extracts, since the compound content of the extracts of both studies fell within the same concentration range. However, in contrast to their results, whereas the inhibitory effect of the ethanol extracts against* S. aureus* and* K. pneumoniae* was higher than that against* E. coli*, our results showed that the inhibitory effect of the ethanol extract was more pronounced against* S. pyogenes* and* E. coli* compared to the other strains (Table 1). Moreover,* K. pneumonia* was the least sensitive strain. These different findings could be attributed to genetic variation between the different bacterial strains or the different radish cultivars (cultivated red radish seeds versus white radish seeds) tested. These findings clearly demonstrate the significant inhibitory potential of cultivated red radish seeds.

### 3.2. Antimicrobial Activity of Seed Fractions and Identification of the Active Compounds

In the current study, an ethanol extract of cultivated red radish seeds (yield range: 5.2–8% w/w) displayed significant antimicrobial activity against gram-positive and gram-negative bacterial strains. In order to explore the active constituents in radish seeds, the ethanol seed extract was further subjected to sequential fractionation, using different solvents with increasing polarity, accompanied with assessing the inhibitory effect of each fraction (bioassay-guided fractionation). The ethyl acetate fraction yielded the greatest inhibitory effect against* E. coli* (Table 2); therefore it was selected for further studies.

In order to further explore the ethyl acetate fraction, this fraction was subjected to fractionation using silica gel column chromatography. Following TLC analysis and pooling of identical fractions, a total of eight different fractions were obtained. Determination of the antimicrobial activity of each of these fractions against* E. coli* revealed that fractions 1 and 2 were the only active fractions, whereas fraction 2 (*R*_*f*_ = 40.3) showed the highest inhibitory effect (Table 3). Fraction 2 (yield 1% w/w, related to the starting material), which was eluted with 1 : 1 (v/v) n-hexane : ethyl acetate, was also more pure than fraction 1 (data not shown) and therefore was selected for further study.

**Figure 1:**
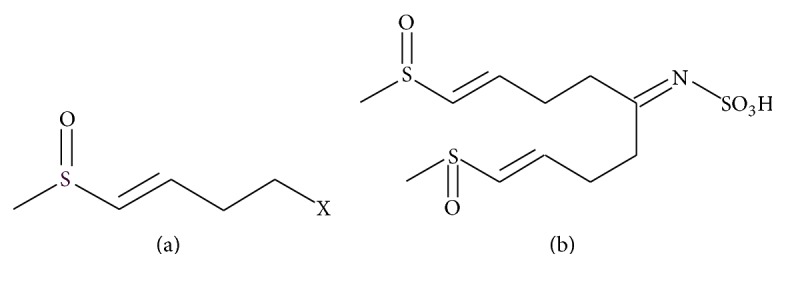
Proposed chemical structure of the active compound of fraction 2. (a) Sulfur-containing chain [4-(methylsulfinyl)but-3-enyl moiety] of the active compound. (b) Determined full structure of the active compound.

In order to identify the active compounds in fraction 2, reversed phase HPLC-MS analysis was used, which showed a single peak (95% purity, data not shown) representing a compound having a calculated mass of 341.04. HRMS of the same fraction confirmed this result and demonstrated that the compound formula is C_11_H_19_NO_5_S_3_. ^1^HNMR (MeOD) recorded the following: *δ* 6.71 (d, *J* = 15.2 Hz); 6.45 (dt *J* = 15.2, 6.5); 3.72 (t, *J* = 6.5); 2.69 (t, *J* = 6.5), suggesting that the major part of the molecule identified by ^1^HNMR was 4-(methylsulfinyl)but-3-enyl (Figure 1(a)). ^13^CNMR recorded the following: 190.0s 137.8d 133.5d 43.6t 40.7q 32.8t (Supplementary data in Supplementary Material available online at http://dx.doi.org/10.1155/2016/9271285). Accordingly, the terminal methyl group, in which the carbon is at 40.7 and its proton is at 2.5 ppm, confirmed that the sulfur in alpha position was oxidized to sulfoxide. Further analysis using ^1^H-^15^N HMBC experiments demonstrated the two correlations to the nitrogen at 86.9 ppm from two different methylene groups at 2.5 ppm and at 3.60 ppm. HMBC experiments that measured carbon-hydrogen correlation revealed a correlation between the carbon at 190 ppm and the hydrogen at 3.6 ppm. Overall, these data suggested that the isolated compound in fraction 2 consisted of two identical sulfur-containing side chains attached to sulfamic acid, and therefore it was identified as [5-methylsulfinyl-1-(4-methylsulfinyl-but-3-enyl)-pent-4-enylidene]-sulfamic acid (MSPSA) (Figure 1(b)). To the best of our knowledge, MSPSA is a new compound.

**Figure 2:**
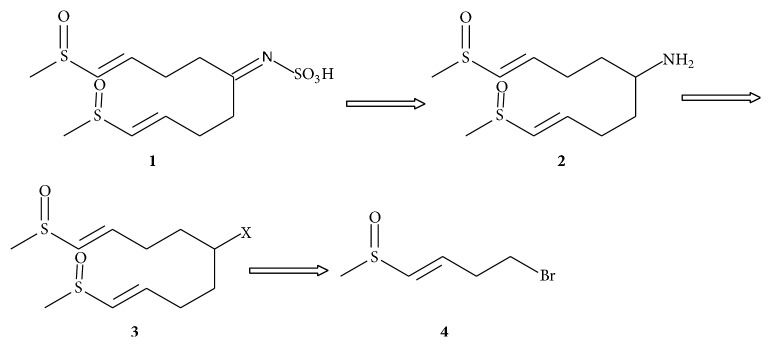
Description of retrosynthesis proposed for MSPSA. The amine** 2** can give the sulfamic acid** 1** by reacting the amine with sulfamic acid. This amine can be prepared from compound** 3**. X in compound** 3** represents leaving group like halogen. The coupling of the monomer** 4** with another monomer gives the dimer** 3**.

Cruciferous plants are well known as main glucosinolates synthesizers. Following injury of the plant cell, thioglucosidases called myrosinases are released resulting in hydrolysis of glucosinolates into various products including ITCs, thiocyanates, and nitriles [6, 26]. Of the approximately 120 described glucosinolates, radish seeds are particularly rich in the aliphatic 4-(methylsulfinyl)but-3-enyl glucosinolate (glucoraphenin) [27–29]. Interestingly, glucoraphanin, its ITC derivative sulforaphene (raphanin), and MSPSA share the same sulfur side chain, that is, 4-(methylsulfinyl)but-3-enyl (the R group of glucosinolates). Aliphatic glucosinolates, such as glucoraphenin, are derived from the amino acid Ala, Leu, Ile, Met, or Val [6, 26]. In the case of glucoraphenin, Met undergoes chain elongation and oxidation of the sulfur atom in the methylthioalkyl side chain leading to methylsulfinylalkyl. Due to the action of myrosinase, D-glucose is released and the unstable aglycone can be converted to isothiocyanates. This step occurs following Lossen rearrangement involving the oxime carbon and the adjacent nitrogen, which also results in release of the sulfate group [6, 26]. Interestingly, MSPA and glucosinolates share the same NSO_3_H group. Thus, it is tempting to speculate that the sulfur side chains of MSPSA are derived from Met in a similar manner to the R group of glucoraphenin and that MSPSA could be a precursor to ITCs. More research is required to gain insight on the biosynthetic pathway of MSPSA. MSPSA could be prepared by retrosynthesis as suggested in Figure 2, thus allowing future modification and preparation of different derivatives to be explored.

Bioactive sulfur compounds, including those containing the 4-(methylsulfinyl)but-3-enyl moiety, seem to be common in radish seeds. During the investigation of the chemical difference between roasted and preroasted radish seeds that are used in traditional Chinese medicine, Zhang et al. [30] have isolated two novel sulfur compounds from preroasted radish seeds identified as* S*-6-(methylsulfinyl)methyl-1,3-thiazinan-2-thione and* O*-ethyl* N*-(*E*)-4-(methylsulfinyl)but-3-enylcarbamothioate. Kim et al. [31] have also isolated seven compounds from radish seeds with anticancer and/or anti-inflammatory activities. Three of these compounds [sinapoyl desulfoglucoraphenin, (*E*)-5-(methylsulfinyl)pent-4-enoxylimidic acid methyl ester, and (*S*)-5-((methylsulfinyl)methyl)pyrrolidine-2-thione] were 4-methylthio-butanyl derivatives and identified as new sulfur compounds. MSPSA, O-ethyl N-(E)-4-(methylsulfinyl)but-3-enylcarbamothioate [30], and the two compounds sinapoyl desulfoglucoraphenin, (*E*)-5-(methylsulfinyl)pent-4-enoxylimidic acid methyl ester, and 5-(methylsulfinyl)-4-pentenenitrile, identified by Kim et al. [31], all contain the 4-(methylsulfinyl)but-3-enyl moiety.

The antimicrobial activity of MSPSA fraction (fraction 2) against the different bacterial strains was similar to that of the crude ethanol extract, suggesting that MSPSA is not the only active compound. The gram-negative strains* E. coli *and* S. typhimurium* were the most sensitive strains. Moreover, the sensitivities of both gram-positive strains* S. pyogenes* and* S. aureus* were the same and similar to that of* K. pneumoniae* (Table 4). In order to quantitatively assess the antimicrobial activity of MSPSA fraction, the MIC values were determined for each of the tested strains. The MIC values ranged between 0.5 and 1 mg/mL, which are 8–64-fold higher than those obtained for chloramphenicol (Table 4), indicating that the antimicrobial effect of MSPSA is relatively less potent compared to that of the control antibiotic (chloramphenicol). Although relatively high, the MIC values of MSPSA are comparable to those reported for other plant extracts, fractions, and isolated compounds [11, 32–34].

Comparison of the inhibitory activity of MSPSA fraction with those of other reported radish fractions containing sulfur compounds, such as ITC-containing fractions, demonstrates that the inhibitory activity of the MSPSA fraction was either less or very similar to ITC-containing fractions, depending on the polarity of the solvent [16]. For example, while in the present study a MIC of 0.5 mg/mL was obtained for the [5-methanesulfinyl-1-(4-methanesulfinyl-but-3-enyl)-pent-4-enylidene]-sulfamic acid fraction, Beevi et al. [16] reported MIC values of 0.016–0.256 mg/mL and of 0.512–1.02 mg/mL for acetone and hexane root extracts containing the 4-(methylthio)-3-butenyl isothiocyanate (MTBITC), respectively. Compared to pure ITCs, the MSPSA fraction was less potent than some aliphatic ITCs, such as allyl isothiocyanate, the most studied ITC, and 4-methylsulfinyl-3-butenyl isothiocyanate (sulphoraphene) [21, 35, 36], but more potent than some aromatic ITCs, such as 4-(*α*-L-rhamnosyloxy)benzyl isothiocyanate and 4-(4′-O-acetyl-*α*-L-rhamnosyloxy)-benzyl isothiocyanate isolated from other plants [37]. Thus, our results indicate that sulfur compounds, including MSPSA, are important antibacterial compounds found in radish seeds.

## 4. Conclusions

During this study, a new antibacterial sulfur compound, MSPSA, was identified. MSPSA consists of 2 sulfur-containing chains similar in structure to the sulfur-containing chains of ITCs. MSPSA was active against medically important gram-negative and gram-positive bacteria with antibacterial activity comparable to that reported for some ITCs. Thus, the obtained results indicate that sulfur compounds, including MSPSA, are important antibacterial compounds found in radish seeds and justify their use in traditional medicine to control infectious diseases.

## Supplementary Material

The structural elucidation of the isolated compound (fraction 2) was performed by spectroscopic methods (1H-NMR, 13C-NMR, and HRMS). High Resolution Mass Spectrum (HRMS) was obtained under the following conditions Single Mass Analysis Tolerance = 5.0 mDa / DBE: min = -1.5, max= 50.0, Element prediction: Off. Number of isotope peaks used for i-FIT = 3. 1H-NMR and 13C-NMR spectra were recorded in deuterated solvents on a Bruker 400 MHz spectrometer.

## Figures and Tables

**Table 1:** Antibacterial activity of radish seed ethanol extract tested against pathogenic bacteria, using the agar well diffusion method. Each value is the mean ± standard deviation of three replicates.

Pathogenic bacteria	Inhibition zone (mm)^a^	
	Extract^b^	Chloramphenicol (10 mg/mL)^c^
*S. pyogenes*	20 ± 2	30 ± 2
*S. aureus*	11 ± 2	15 ± 1
*E. coli*	19 ± 0	27 ± 2
*K. pneumoniae*	9 ± 2	16 ± 3
*S. typhimurium*	16 ± 3	29 ± 2

^a^Inhibitory zones in mm, including diameter of the well (6.0 mm); mean ± standard deviation of three replicates.

^b^The concentration of the extract was 130–200 mg/mL; 70–80 *μ*L was added to each well.

^c^15 *μ*L was added to each well.

**Table 2:** Antibacterial activity of seed extract fractions tested against *E. coli* determined by the agar well diffusion method. Each value is the mean ± standard deviation of three replicates.

Fraction (10 mg/well^a^)	Inhibition zone (mm)^b^
Ethyl acetate	27 ± 2
Hexane	12.5 ± 1
Water (remaining fraction)	—
Chloramphenicol^c^	27 ± 2

^a^10 mg of dried extract was dissolved in 100 *μ*L of 15% methanol and the whole volume added to the well. —: no inhibition.

^b^Inhibitory zones in mm, including diameter of the well (6.0 mm).

^c^15 *μ*L of 10 mg/mL was added to each well.

**Table 3:** Antibacterial activity tested against *E. coli* of the eight ethyl acetate fractions separated using silica gel column chromatography and determined using the agar well diffusion method. Each value is the mean ± standard deviation from three replicates.

Fraction (2 mg/well^a^)	Inhibition zone (mm)^b^
Fraction 1	22 ± 1
Fraction 2	26 ± 1
Fractions 3–8	—
Chloramphenicol^c^	27 ± 2

^a^2 mg of dried fraction was dissolved in 100 *μ*L of 15% methanol and the whole volume added to the well.

^b^Inhibitory zones in mm, including diameter of the well (6.0 mm). —: no inhibition.

^c^15 *μ*L of 10 mg/mL was added to each well.

**Table 4:** Antibacterial activity of fraction 2 tested against various pathogenic bacteria. Each value is the mean ± standard deviation of three replicates.

Bacteria	Inhibition zone (mm)^a^		MIC (mg/mL)	
	MSPSA fraction (2 mg/well)^b^	Chloramphenicol (0.16 mg/well)	MSPSA fraction	Chloramphenicol
*S. pyogenes*	13 ± 2	15 ± 2	1	0.0625
*S. aureus*	13 ± 2	15 ± 1	0.5	0.0625
*E. coli*	25 ± 2	29 ± 2	0.5	0.0078
*K. pneumoniae*	15 ± 1	15 ± 1	1	0.0625
*S. typhimurium*	19 ± 2	27 ± 2	0.5	0.0078

^a^2 mg of dried fraction was dissolved in 100 *μ*L of 15% methanol and the whole volume was added to the well.

^b^Inhibitory zones in mm, including diameter of the well (6.0 mm).
