# Supplementary material for: Identification of a New Antibacterial Sulfur Compound from Raphanus sativus Seeds
Source: Evid Based Complement Alternat Med. 2016 Oct 3;2016:9271285. doi: 10.1155/2016/9271285 (PMC5066007; doi:10.1155/2016/9271285)
Supplement: Supplementary file 1 — The structural elucidation of the isolated compound (fraction 2) was performed by spectroscopic methods (1H-NMR, 13C-NMR, and HRMS). High Resolution Mass Spectrum (HRMS) was obtained under the following conditions Single Mass Analysis Tolerance = 5.0 mDa / DBE: min = -1.5, max= 50.0, Element prediction: Off. Number of isotope peaks used for i-FIT = 3. 1H-NMR and 13C-NMR spectra were recorded in deuterated solvents on a Bruker 400 MHz spectrometer. [file 9271285.f1.pdf]

Supplementary data

HRMS of fraction 2 demonstrating the calculated molecular mass (341.04) and the formula (C11, H19, N, O5, S3) of the compound.

Elemental Composition Report

Single Mass Analysis
Tolerance = 20.0 mDa / DBE: min = -1.5, max = 50.0
Element prediction: Off
Number of isotope peaks used for i-FIT = 3

Monoisotopic Mass, Odd and Even Electron Ions
48 formula(e) evaluated with 6 results within limits (all results (up to 1000) for each mass)
Elements Used:
C: 10-15 H: 17-20 N: 0-2 O: 0-6 S: 0-3

13-Jun-2013
cli_5716p 169 (1.459) Cm (151:256-87:117)
sample
Galilee Society
TOF MS ES+
7.40e+004

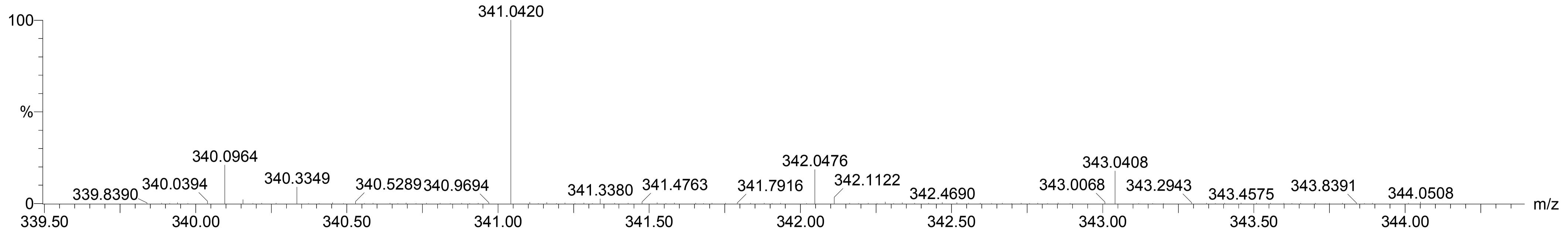

Mass	Calc. Mass	mDa	PPM	DBE	i-FIT	Formula
Minimum:				-1.5		
Maximum:		20.0	10.0	50.0		
341.0420	341.0425	-0.5	-1.5	3.0	233.3	C11 H19 N O5 S3
	341.0452	-3.2	-9.4	7.5	132.8	C14 H17 N2 O2 S3
	341.0340	8.0	23.5	7.5	283.8	C15 H17 O3 S3
	341.0517	-9.7	-28.4	7.5	972.8	C15 H17 O5 S2
	341.0300	12.0	35.2	3.5	716.0	C10 H17 N2 O5 S3
	341.0578	-15.8	-46.3	7.0	693.0	C15 H19 N O2 S3

^{1}HNMR of fraction 2

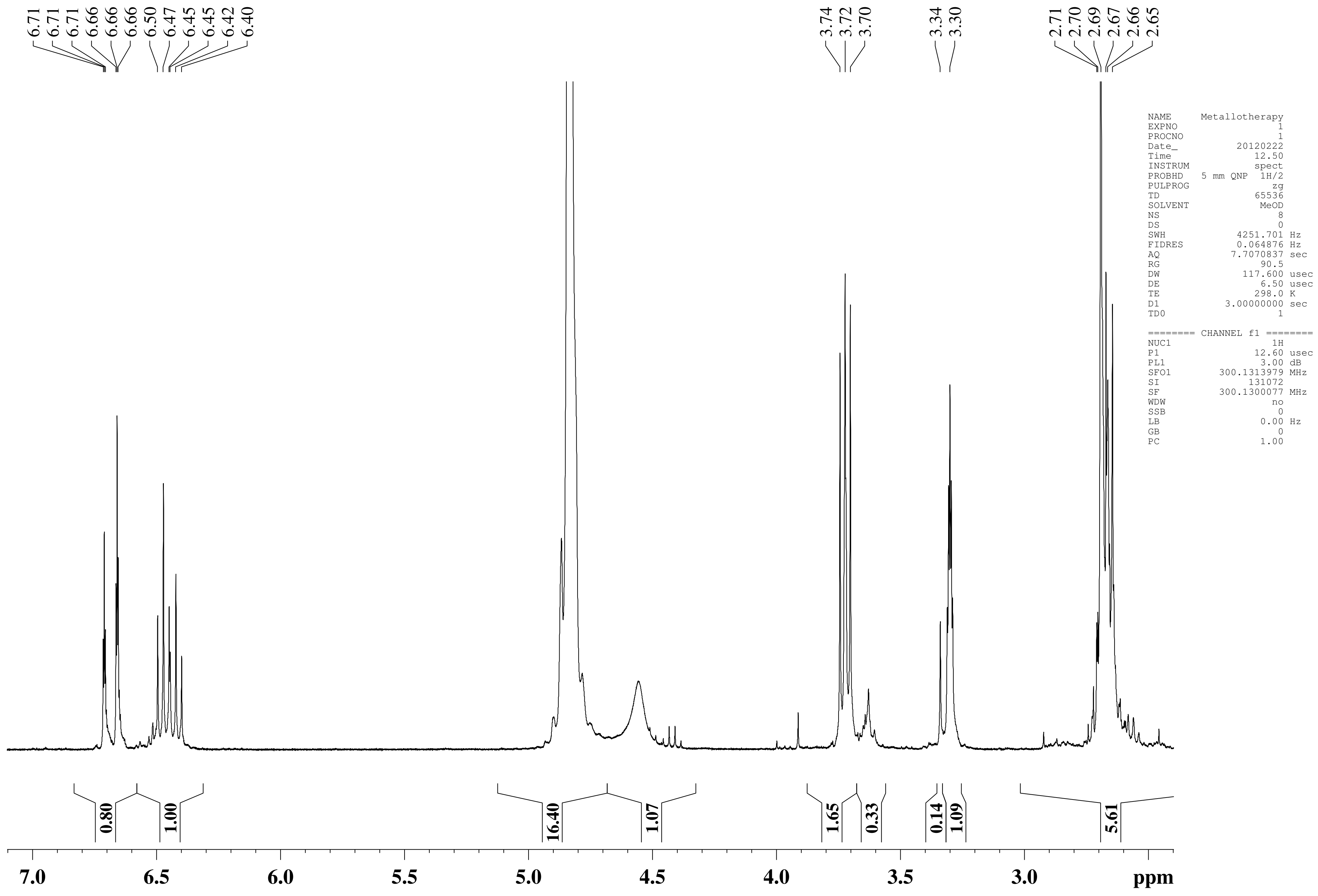
6.71
6.71
6.71
6.66
6.66
6.66
6.50
6.47
6.45
6.45
6.42
6.40
3.74
3.72
3.70
3.34
3.30
2.71
2.70
2.69
2.67
2.66
2.65
NAME Metallotherapy
EXPNO 1
PROCNO 1
Date_ 20120222
Time 12.50
INSTRUM spect
PROBHD 5 mm QNP 1H/2
PULPROG zg
TD 65536
SOLVENT MeOD
NS 8
DS 0
SWH 4251.701 Hz
FIDRES 0.064876 Hz
AQ 7.7070837 sec
RG 90.5
DW 117.600 usec
DE 6.50 usec
TE 298.0 K
D1 3.00000000 sec
TD0 1
======== CHANNEL f1 ========
NUC1 1H
P1 12.60 usec
PL1 3.00 dB
SFO1 300.1313979 MHz
SI 131072
SF 300.1300077 MHz
WDW no
SSB 0
LB 0.00 Hz
GB 0
PC 1.00
0.80
1.00
16.40
1.07
1.65
0.33
0.14
1.09
5.61
7.0
6.5
6.0
5.5
5.0
4.5
4.0
3.5
3.0
ppm

^{13}CNMR of fraction 2

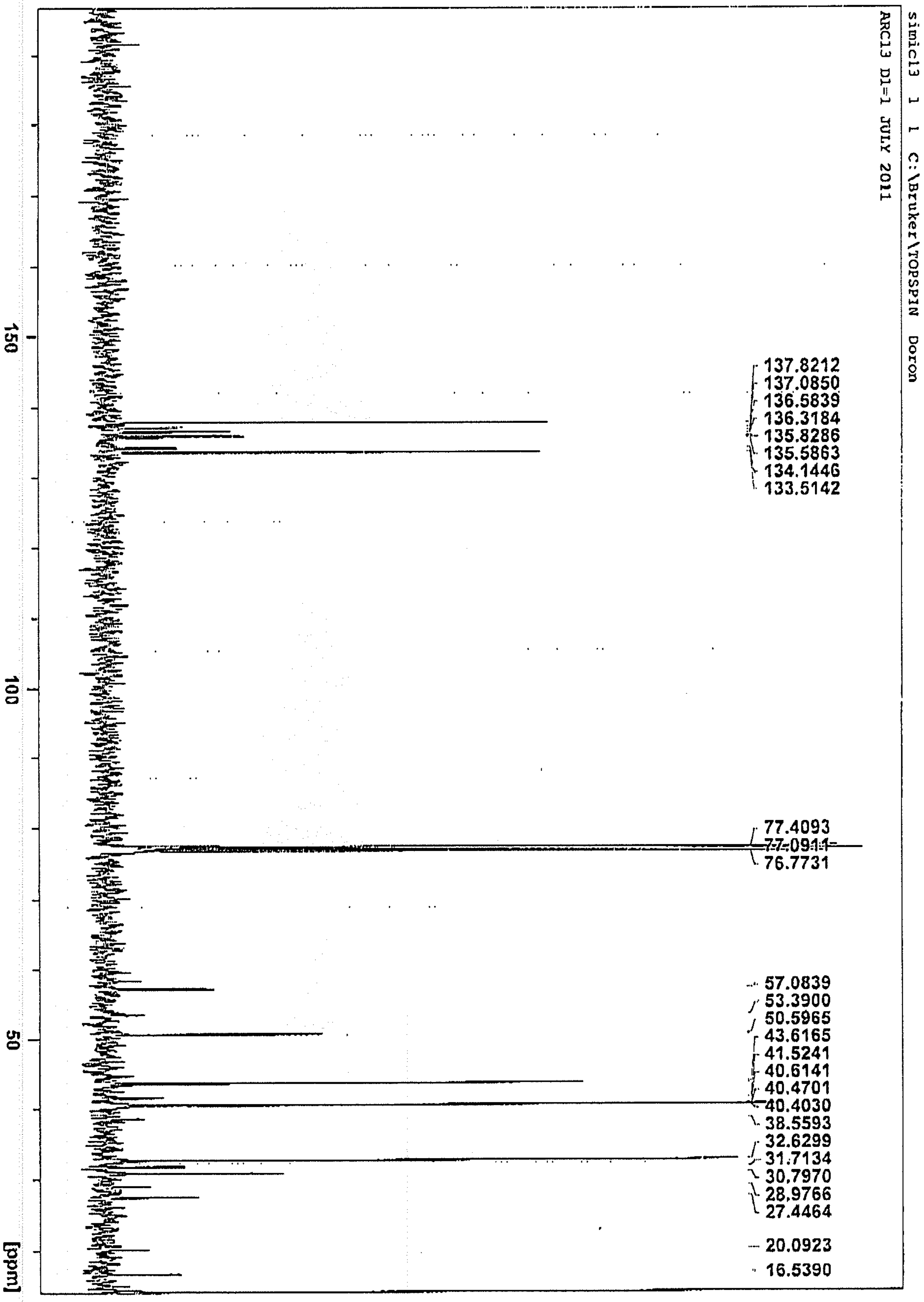
simic13 1 1 C:\Bruker\TOPSPIN Doron
ARC13 D1=1 JULY 2011
137.8212
137.0850
136.5839
136.3184
135.8286
135.5863
134.1446
133.5142
77.4093
77.0911
76.7731
57.0839
53.3900
50.5965
43.6165
41.5241
40.6141
40.4701
40.4030
38.5593
32.6299
31.7134
30.7970
28.9766
27.4464
20.0923
16.5390
150
100
50
[ppm]
